# Fibrolipoma of the lower lip: A case report and review of the literature

**DOI:** 10.1097/MD.0000000000038543

**Published:** 2024-06-21

**Authors:** Dengshun Wang, Hongwei Yu, Tong Gao, Haibin Lu, Yu’e Wang

**Affiliations:** a Department of Oral and Maxillofacial Surgery, Zhongshan Hospital Affiliated to Dalian University, Dalian, Liaoning Province, People’s Republic of China; b School of Stomatology, Dalian University, Dalian, Liaoning Province, People’s Republic of China.

**Keywords:** case report, clinical features, intraoral lesions, lip, lipoma

## Abstract

**Background:**

Fibrolipoma of the lower lip is an uncommon condition with limited documentation in the literature. This paper provides updated insights into oral and maxillofacial lipomas through a detailed case report and comprehensive literature review, discussing clinical features, pathogenesis, diagnostic approaches, histopathology, and therapeutic strategies.

**Case presentation:**

A 54-year-old female presented with a painless, enlarging mass on the inner aspect of her right lower lip, first noticed 2 years prior. The mass, now the size of a peanut, interfered with her eating and speech. Physical examination revealed a 2.0 × 2.5 × 1.0 cm mass beneath the mucous membrane of the right lower lip. It was firm, well-demarcated, and mobile. Surgical excision was performed, and histopathological analysis confirmed the diagnosis of a lower lip fibrolipoma. The lesion was successfully removed without recurrence.

**Conclusion:**

Lipomas in the oral and maxillofacial regions are rare, slow-growing benign tumors, particularly within the lips. Although their diagnosis is straightforward based on clinical presentation, histopathological confirmation is essential. Surgical resection remains the treatment of choice, with excellent prognostic outcomes.

## 1. Introduction

Lipomas are the most prevalent benign tumors, occurring throughout the body but predominantly on the trunk.^[1]^ Approximately 15% to 20% of lipomas arise in the head and neck area, with oral lipomas being relatively rare, constituting only 1% to 4% of all cases. Currently, there is no definitive data on the incidence of intra-oral lipomas at specific sites. Nevertheless, a 14-year retrospective study analyzing the clinical and morphological characteristics of oral lipomas identified the buccal mucosa as the most frequently affected area, followed by the tongue, with the lower lip being an uncommon site.^[2]^

The histopathological spectrum of oral and maxillofacial lipomas includes traditional lipomas, fibrolipomas, spindle cell lipomas, salivary lipomas, osteolipomas, chondrolipomas, and intramuscular lipomas. Fibrolipoma, distinguished by a dense network of fibrous collagen and mature fat cells, is classified by the World Health Organization as a rare histological subtype of traditional lipomas.^[3]^

The occurrence of fibrolipoma in the lower lip is exceedingly rare. This paper provides a comprehensive review of lower lip fibrolipoma cases treated at our hospital from 1982 to 2023 and examines relevant studies on labial lipoma from the CNKI and PubMed databases. This extensive exploration discusses the clinical manifestations, pathogenesis, histological characteristics, differential diagnosis, and treatment strategies for labial lipoma.

## 2. Case report

### 2.1. Patient information

The patient, a 54-year-old female, was admitted to the hospital presenting with a mass in the right lower lip that had been growing for 2 years and recently began affecting her speech and eating abilities. She reported no previous lip trauma, no habit of lip biting, and no history of smoking or prior surgeries.

### 2.2. Clinical findings

During the physical examination, a 2.0 × 2.5 × 1.0 cm mass was observed under the mucosa of the right lower lip. It was the same color as the surrounding mucosa, had a smooth surface, soft texture, clear boundaries, good mobility, and was not tender. The postural test was negative; with the patient head in a low position, there was no hyperemia or swelling in the lesion area, and no deviation from its appearance in a normal position. The mass was tentatively diagnosed as a benign soft tissue tumor of the lip, pending further diagnostic assessment.

### 2.3. Therapeutic intervention and diagnostic assessment

**Figure 1.**
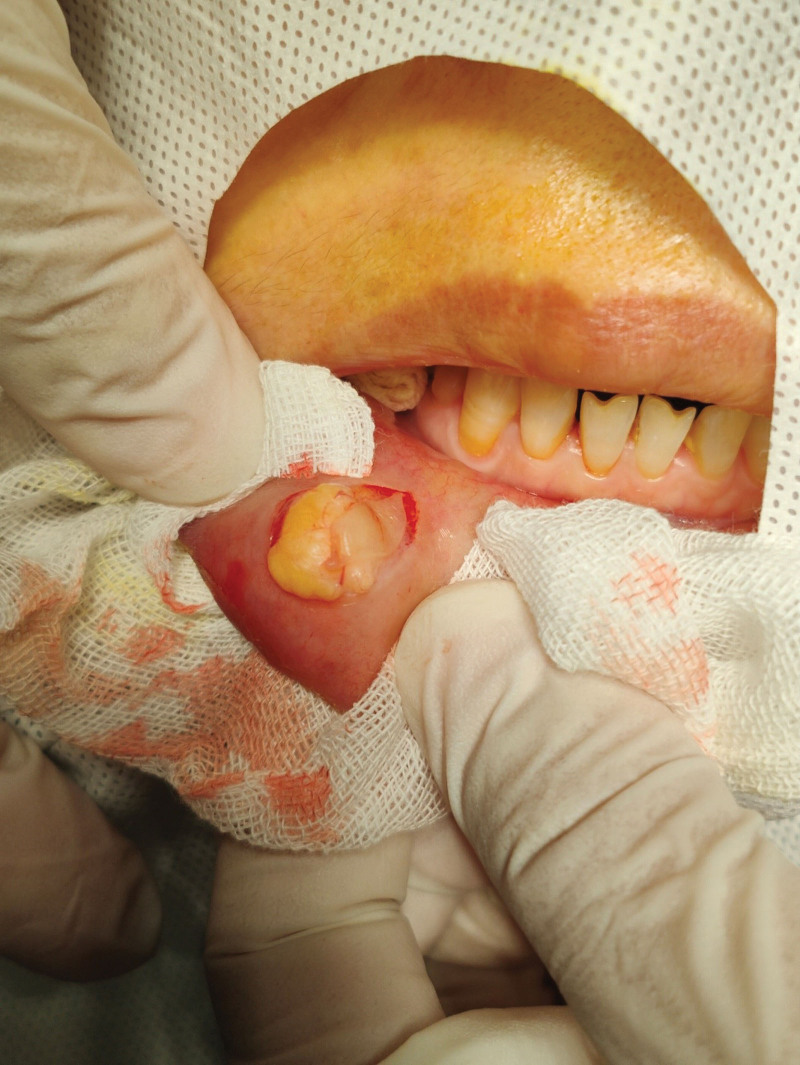
Intraoperative photograph. The submucosal lesion is yellow, smooth, soft, and measures 2.0 × 2.5 × 1.0 cm.

**Figure 2.**
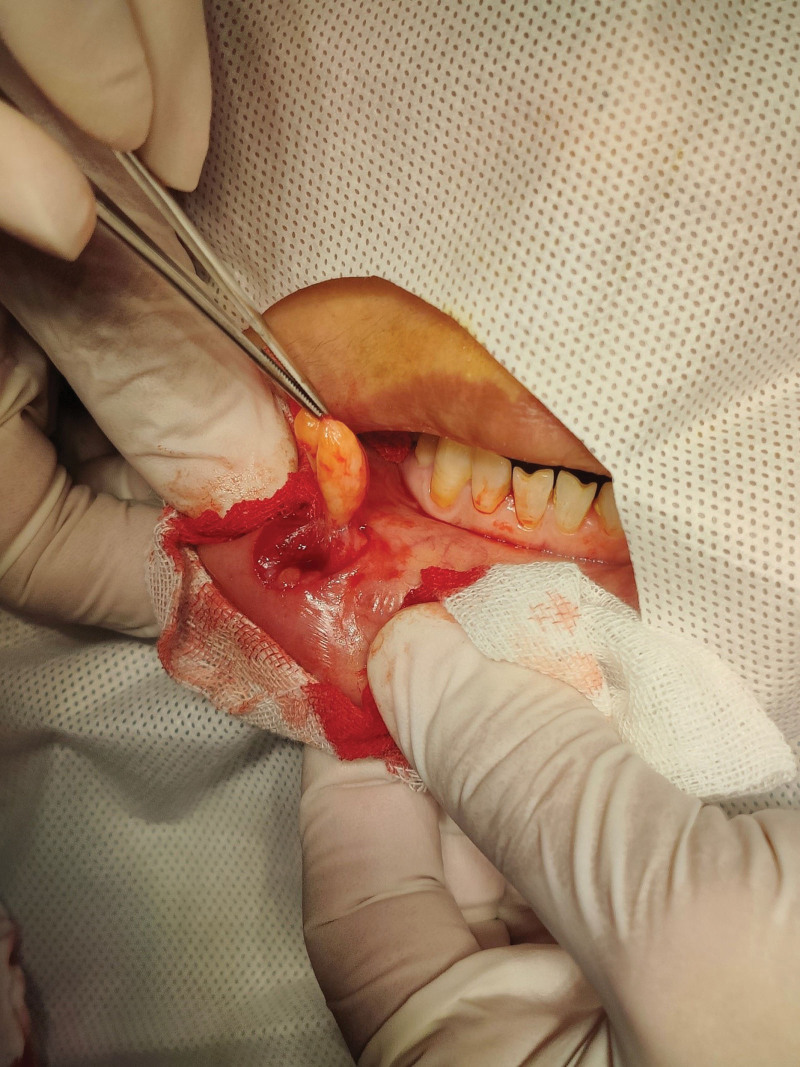
Intraoperative photograph. The lesion is located above the muscular layer with clear boundaries.

**Figure 3.**
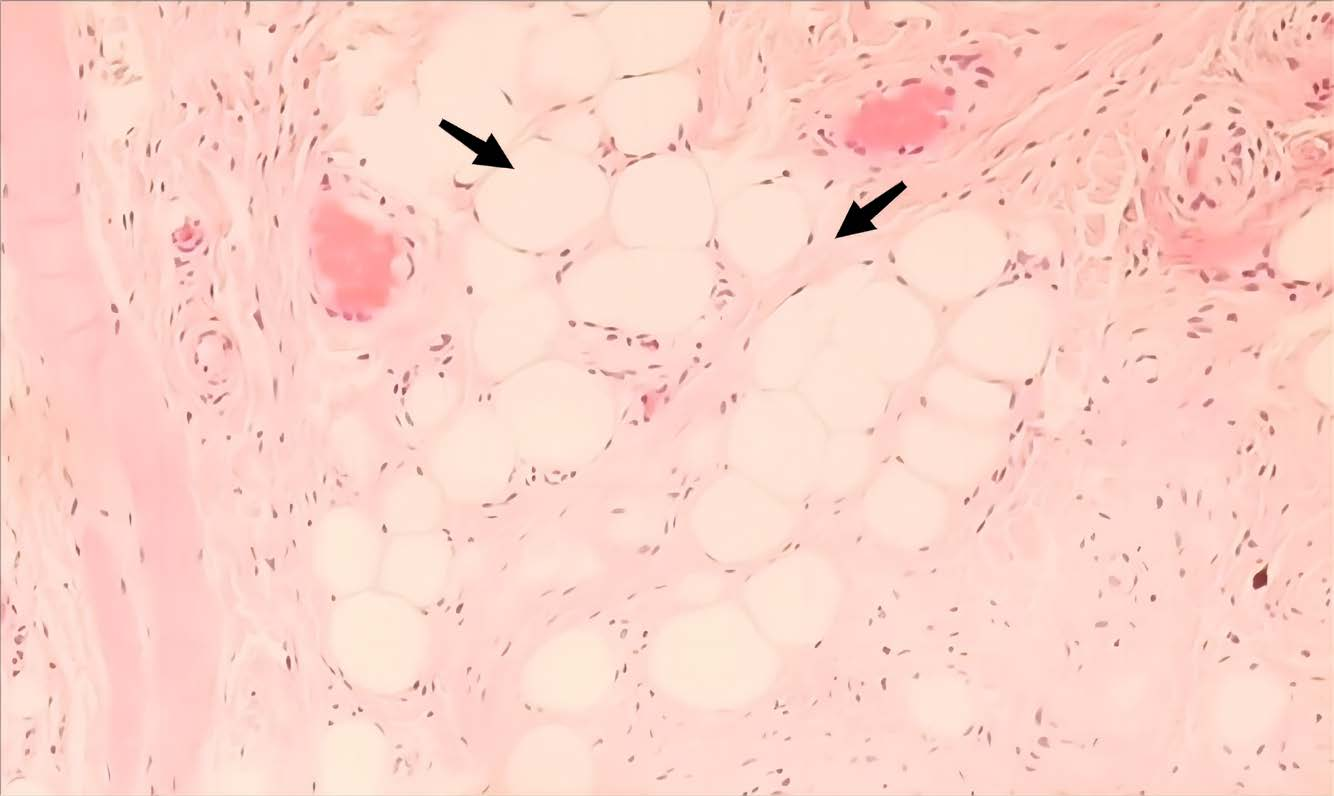
Pathology. The biopsy shows fibrolipoma, with a large amount of fibrous and adipose tissue (H&E, ×100).

The mass was excised under local anesthesia. It measured 2.0 cm in diameter, was soft, yellow, and clearly demarcated (see Fig. 1). The tumor was located superficial to the lip muscle (see Fig. 2). Histological examination confirmed the presence of fibrolipoma, characterized by abundant fibrous and adipose tissue (see Fig. 3).

### 2.4. Follow-up and outcomes

**Figure 4.**
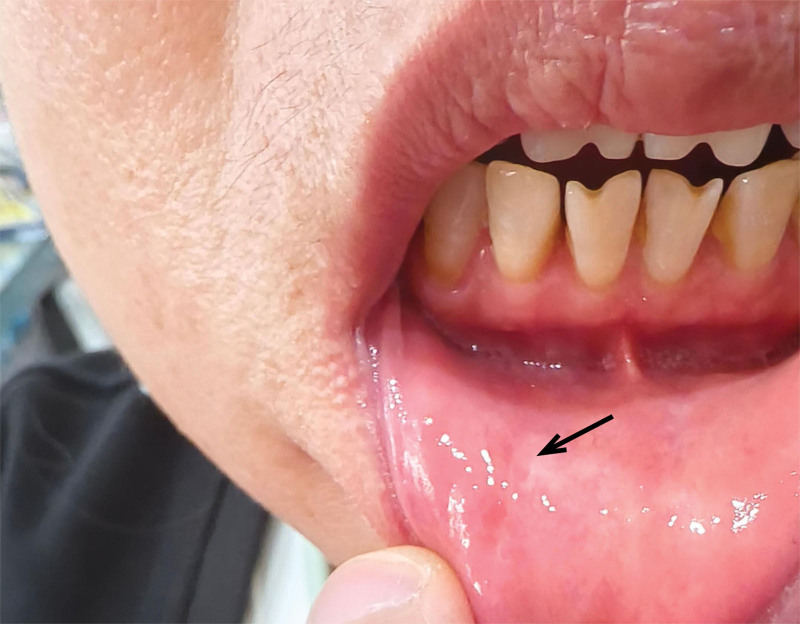
Postoperative follow-up. Three months after the surgery, the patient returned for a follow-up visit and showed good recovery without recurrence.

The patient showed good recovery during the follow-up period after discharge, with no recurrence observed (see Fig. 4).

## 3. Literature review

CNKI and PubMed databases were searched in both Chinese and English, covering the period from January 1982 to November 2023. The Chinese search terms included “Maxillofacial lipoma” and “lower lip lipoma,” while the English search terms were “Maxillofacial lipoma,” “lip lipoma,” and “oral lipoma.” Inclusion criteria were: Diagnosis of lip lipoma; availability of complete clinical data, such as gender, age, site, size, pathological type, and treatment plan; exclusion of lipomas at other sites. A total of 64 studies were reviewed, encompassing 671 patients, of whom 358 were male, 312 were female, and 1 was identified as genderless. The cases spanned various locations including the buccal area, floor of mouth, gums, tongue, submaxillary region, palate, parotid gland, cheek, frontotemporal region, neck, nasal septum, and mandibular canal. Among these, 14 cases met the inclusion criteria, with 12 reported in international literature and 2 in domestic publications. In total, 21 cases involving lipoma were reported, 19 of which were documented in international literature and only 2 in domestic sources (see Table 1). Notably, Table 1 includes 2 cases of lip fibrolipoma. Compared to the case reported in this study, no significant differences were observed in the age, sex, and location of the lesions, except for their size, which may vary depending on the duration of the lesion.

**Table 1:** Lip lipomas published in domestic and international literature from 1982 to 2023.

Author	Country	Yr	Age/sex	Location	Size	Pathology	Treatment
Ashika et al^[4]^	IND	2023	28/M	Upper lip	2.5 × 2.0 cm	Lipoma	Excision
Dastoor et al^[5]^	IND	2018	11/M	Upper lip	1.0 × 1.0 cm	Fibrolipoma	Excision
Aita et al^[6]^	BRA	2017	75/F	Lower lip	4.5 × 3 × 2.5 cm	Lipoma	Excision
Morais et al^[7]^	BRA	2009	6M/M	Upper lip	3.7 cm	Lipoma	Excision
Aguiar et al^[8]^	BRA	2009	35/F 38/M 57/M 56/F	Lower lip Lower lip Lower lip Lower lip	0.5 cm 1.0 cm 1.2 cm 2.1 cm	Spindle-cell lipoma Sialolipoma Lipoma Lipoma	Excision Excision Excision Excision
Zussman et al^[9]^	USA	1988	43/M	Lower lip	2.0 cm	Lipoma	Excision
De Visscher et al^[10]^	NLD	1982	41/M 66/F 68/M 44/M 63/M	Lower lip Lower lip Lower lip Lower lip Upper lip	0.8 cm 1.5 cm 0.7 cm 1.5 cm 1.0 cm	Lipoma Fibrolipoma Lipoma Lipoma Fibrolipoma	Excision Excision Excision Excision Excision
Correll et al^[11]^	USA	1982	48/M	Lower lip	1.5 cm	Lipoma	Excision
Turner et al^[12]^	USA	1956	74/M	Lower lip	3.0 cm	Lipoma	Excision
Kumar et al^[13]^	IND	2014	28/M	Lower lip	2.5 × 2.0 cm	Lipoma	Excision
Perez et al^[14]^	BRA	2007	42/M	Lower lip	1.5 cm	Fibrolipoma	Excision
Panagiotis et al^[15]^	GRC	2000	65/F	Lower lip	1.5 cm	Lipoma	Excision
Xu et al^[16]^	CHN	2020	44/M	Lower lip	1.0 × 1.0 cm	Lipoma	Excision
Wu et al^[17]^	CHN	2016	31/F	Lower lip	1.0 × 0.8 cm	Lipoma	Excision

## 4. Discussion

Roux first described oral lipomas in an 1848 review of alveolar masses, which he termed “yellow epulis.”^[18]^ Lipoma is a commonly encountered mesenchymal tumor, yet oral lipoma remains relatively rare, with a prevalence of 1 in 5000.^[19]^ Fibrolipoma, a microscopic variant of lipoma, is histologically characterized by mature fat cells segregated into lobules by fibrous tissue.^[5]^ The typical locations for fibrolipomas mirror those of classical lipomas, largely dependent on the concentration of adipose tissue. These tumors most frequently manifest in the buccal mucosa, followed by the tongue, and are seldom found in the lower lip.^[4]^ Previous research has not delineated the proportion of lipomas occurring specifically in the upper versus lower lip. However, according to a literature review, only 4 of 21 documented cases of lipomas were located in the upper lip, suggesting a lower incidence there compared to the lower lip.^[4,5,7,10]^ Chronic stimulation, such as that from repeated mechanical irritation, may promote adipose tissue proliferation,^[20]^ potentially accounting for the higher prevalence of lipoma in the lower lip. Due to the small sample size, limited database sources for the literature review, and potential biases, the results of this study should be interpreted with caution.

Oral lipomas and their histological variants are observed across all age groups but are predominantly seen in individuals between 40 and 60 years of age, with a higher prevalence among women for fibrolipomas.^[2]^ An analysis involving 1 patient from our hospital and a review of 671 cases cited in this study revealed that the age of onset ranged from as young as 4 months to as old as 92 years, with a peak incidence between 40 and 60 years. This pattern aligns with findings reported in both domestic and international research. Additionally, Aguiar de Freitas et al noted that the incidence of fibrolipoma is equally distributed between the fifth and seventh decades of life.^[8]^

The literature presents varying perspectives on the gender distribution of oral and maxillofacial lipomas. Furlong, Taira et al contend that these tumors are more prevalent in males,^[21,22]^ whereas Pires et al assert a higher likelihood in females.^[23]^ Conversely, da Cruz Perez, Panagiotis, et al along with most scholars, maintain that there is no significant gender difference in the occurrence of these lipomas.^[14,15]^ Our analysis, which included 1 patient from our hospital and a review of 671 cases documented in the literature, led to the exclusion of 1 case due to unknown gender. The remaining cases comprised 358 males and 312 females, resulting in a male-to-female ratio of 1.147:1. This finding indicates no significant gender difference, aligning with the consensus in most studies.

Clinically, oral lipomas are typically characterized as painless, slow-growing masses that only become noticeable when they reach a significant size. The tumor movement, color, and texture may vary according to the depth of the tumor and the amount and distribution of the fibrous tissue within different anatomical sites.^[6,9,11,24]^ Although most oral lipomas are asymptomatic, cases of fibrolipomas causing mucosal ulcerative changes have been documented.^[25]^ In the oral and maxillofacial region, lipomas are usually solitary; however, bilateral involvement or multiple occurrences may suggest a clinical diagnosis of benign symmetrical lipomatosis, multiple spindle cell lipomas, or multiple classical lipomas.^[26]^ In this study, no unique characteristics were observed in the 25 cases of lip lipoma.

The etiology of fibrolipoma and simple lipoma remains elusive. Current studies suggest the following potential mechanisms for their pathogenesis: Congenital factors; endocrine disorders; fibroma degeneration; adipocyte maturation.^[27,28]^ Notably, the post-traumatic lipoma of the lower lip reported by Turner et al in 1956 highlighted trauma as a possible inducer of lipoma.^[29]^ Tewfik et al documented a case of subcutaneous lipoma following a parietal fracture, outlining the potential mechanisms for posttraumatic lipoma development: Disruption of the fibrous septum and anchoring connections between the skin and deep fascia, leading to adipose tissue proliferation; local inflammation secondary to trauma, which may promote preadipocyte differentiation and maturation.^[12]^ Furthermore, Koh et al identified the use of tamoxifen and subcutaneous injections of phosphatidylcholine and deoxycholate as potential risk factors for lipoma formation.^[30]^ In conclusion, the pathogenesis of lipoma is multifaceted, and in cases of lip lipoma, chronic irritation from repeated chewing may play a contributory role.

Although lipomas are relatively rare in the oral cavity, their clinical diagnosis is typically straightforward. However, due to its fibrous content, a fibrolipoma may be clinically mistaken for a fibroma based on texture. Histologically, fibrolipoma is characterized by a high content of collagen and connective tissue, which can cause it to adhere to surrounding tissues and form focal pseudo-infiltrations. This sometimes necessitates differentiation from malignant infiltrations, such as liposarcoma.^[28]^ Lip lipomas are often misdiagnosed due to their location, as they can be mistaken for mucinous gland cysts, venous malformations, lymphatic vessel malformations, and other common masses in the lower lip, thus requiring careful differential diagnosis. Additionally, the presence of minor salivary glands in the area necessitates the consideration of basal cell adenomas as a potential diagnosis.^[31]^

Histologically, lipomas are classified based on the composition and proportion of various tissue types, including fibrous connective tissue, spindle cells, mitotically active atypical cells, mature blood vessels, mucoid matrix, salivary acinar structures, and mature adipocytes. The classifications include classic lipomas, fibrolipomas, angiolipomas, spindle cell lipomas, pleomorphic lipomas, myxoid lipomas, salivary gland lipomas, and intramuscular lipomas.^[13,16,17]^

Based on our literature review, we identified reports of classic lipomas, fibrolipomas, spindle cell lipomas, salivary gland lipomas, and lipoblastoma of the lip, with the latter 3 being relatively rare. Spindle cell lipomas, characterized by a mix of mature adipocytes and spindle cell proliferation, may be misdiagnosed as conventional lipomas, fibrolipomas, or myxoid lipomas. This misidentification can occur due to varying proportions and the resemblance of spindle cells to fibrous tissue and myxoid matrix.^[2]^ Salivary gland lipomas are distinguished by mature adipose tissue interspersed with salivary gland tissue, occasionally featuring epithelial islands at the tumor periphery.^[32]^ Under microscopic examination, lipoblastoma presents a mixture of adipoblasts and adipocytes, necessitating clinical differentiation from Fordyce spots, nevus of superficial lipoma, and foreign body granuloma.^[33]^

The standard treatment for lipomas and their histological variants is local resection, with prognosis generally favorable across types, as recurrences are rare, except in cases of intramuscular lipomas.^[34]^ Other studies have indicated that for lipomas smaller than 2.5 cm, a daily repeated injection of a 1:1 lidocaine and triamcinolone mixture, ranging from 1 to 3 cm, is effective; for tumors measuring 4 to 10 cm or larger, 16# needle liposuction is recommended.^[13,35]^ Laser surgery has also been proven effective for treating lip fibrolipoma, offering a valuable alternative to traditional surgery due to advantages such as no intraoperative bleeding, minimal tissue scarring, rapid patient recovery, and preservation of surrounding tissues.^[36]^ Immunohistochemical analysis of proliferating cell nuclear antigen and Ki-67 expression is useful for assessing the proliferative activity of lipomas, with Ki-67 expression serving as a predictor of tumor recurrence or malignant transformation.^[20]^ Studies have also shown that fibrolipomas exhibit higher Ki-67 expression compared to classical lipomas and other variants, underscoring the need for regular follow-up to monitor the local condition of the patient.

## 5. Conclusion

Fibrolipoma of the lower lip is a rare entity, and this case study enhances our comprehensive understanding of such neoplasms. Through an extensive analysis of the literature, we have summarized the diagnostic and treatment approaches for oral and maxillofacial lipomas. Although clinical manifestations can offer preliminary diagnostic clues, pathological examination is crucial for definitive confirmation. Surgical resection is the preferred treatment method, typically resulting in favorable prognostic outcomes.

## 6. Key message/recommendations

Although rare, clinicians should consider the possibility of a lipoma when encountering abnormal masses on the lower lip.

Early diagnosis and prompt surgical treatment are crucial for patients with lower lip lipomas to avoid the growth of the mass, which can affect aesthetics and oral function.

## Author contributions

**Data curation:** Hongwei Yu.

**Investigation:** Tong Gao.

**Resources:** Yu’e Wang.

**Supervision:** Haibin Lu.

**Writing – review & editing:** Dengshun Wang.
